# Dental Caries Risk Assessment in Children 5 Years Old and under via Machine Learning

**DOI:** 10.3390/dj10090164

**Published:** 2022-09-01

**Authors:** Seyed-Ali Sadegh-Zadeh, Ali Rahmani Qeranqayeh, Elhadj Benkhalifa, David Dyke, Lynda Taylor, Mahshid Bagheri

**Affiliations:** Department of Computing, Staffordshire University, Stoke-on-Trent ST4 2DE, UK

**Keywords:** caries prediction, dental medicine, dental caries, artificial intelligence, diagnostic prediction

## Abstract

Background: Dental caries is a prevalent, complex, chronic illness that is avoidable. Better dental health outcomes are achieved as a result of accurate and early caries risk prediction in children, which also helps to avoid additional expenses and repercussions. In recent years, artificial intelligence (AI) has been employed in the medical field to aid in the diagnosis and treatment of medical diseases. This technology is a critical tool for the early prediction of the risk of developing caries. Aim: Through the development of computational models and the use of machine learning classification techniques, we investigated the potential for dental caries factors and lifestyle among children under the age of five. Design: A total of 780 parents and their children under the age of five made up the sample. To build a classification model with high accuracy to predict caries risk in 0–5-year-old children, ten different machine learning modelling techniques (DT, XGBoost, KNN, LR, MLP, RF, SVM (linear, rbf, poly, sigmoid)) and two assessment methods (Leave-One-Out and K-fold) were utilised. The best classification model for caries risk prediction was chosen by analysing each classification model’s accuracy, specificity, and sensitivity. Results: Machine learning helped with the creation of computer algorithms that could take a variety of parameters into account, as well as the identification of risk factors for childhood caries. The performance of the classifier is almost unbiased, making it generalizable. Among all applied machine learning algorithms, Multilayer Perceptron and Random Forest had the best accuracy, with 97.4%. Support Vector Machine with RBF Kernel (with an accuracy of 97.4%) was better than Extreme Gradient Boosting (with 94.9% accuracy). Conclusion: The outcomes of this study show the potential of regular screening of children for caries risk by experts and finding the risk scores of dental caries for any individual. Therefore, in order to avoid dental caries, it is possible to concentrate on each individual by utilizing machine learning modelling.

## 1. Introduction

Dental caries is the most common dental disease among children and the single most common chronic childhood disease, with considerable economic and quality-of-life burdens [1]. It is a dynamic, multifactorial, but preventable disease with well-known participating factors. It is caused by the dissolution of teeth by acid production due to the metabolism of carbohydrates by certain bacteria [2]. Its prevalence is thought to have increased recently in children aged 2–5 years globally, making this age group a global priority action area.

If left untreated, dental caries can lead to pain, discomfort, failure to thrive, reduced quality of life, and tooth loss [3]. Severe dental caries among children has a significant negative impact on family life, as well. Parents of children with severe dental caries have been shown to take more time off work, report that the child needed more attention, felt guilty, felt stressed, have normal activities disrupted, and have sleep disrupted [4]. Therefore, it is important to detect young patients at risk and provide them with thorough prevention measures and a tailored approach to care.

Many caries risk and prediction models have been introduced and tested [5]. A risk model is used to identify one or more risk factors for the disease, while a prediction model identifies individuals at high risk [6]. Three main approaches have been identified for caries risk assessment:

(a) Past caries experience: it is believed that those who develop caries in the first years of life tend to develop more lesions; however, this does not specify the particular risk factors [7,8].
(b) Socioeconomic factors: people living in certain districts or belonging to certain ethnic or religious groups may be ‘risk individuals’.
(c) Biological factors such as diet, host, and bacteria could be used in order to predict dental caries, as high sugar intake, low fluoride exposure, and high counts of cariogenic bacteria in saliva/plaque contribute to dental caries [1,9].

In children, the strongest predictor of caries incidence is previous caries experience and present caries. Other predictors include extended breastfeeding, high counts of salivary [10] cariogenic bacteria, poor oral hygiene habits, low/no fluoride exposure, high sugar intake, parents’/carers’ low socioeconomic status, and smoking, as social factors usually explain the reason for neglected oral hygiene and increased sugar consumption [11,12,13]. Several reliable studies [14,15,16] indicate that second-hand smoke was connected to an increased risk of increases in caries in children, but these connections may be complicated by unmeasured lifestyle factors such as dental cleaning.

Cariogram [5], CAMBRA [17], PreVisor, NUS-CRA, and CAT are the most well-known caries risk assessment models that have been studied. It has been shown that multivariate models are generally better than their single-predictor counterparts [17]. We believe that an acceptable model is the one that considers the most important variables and is user/patient-friendly, especially when dealing with young children.

Machine learning is a branch of artificial intelligence. It provides a strategic approach to the development of automated, sophisticated, and objective algorithmic techniques for data analysis [18,19]. In machine learning, training datasets are used to train classification algorithms. These algorithms are able to automatically generate rules to perform data mining or predict the future outcome of features by identifying patterns in the training data. These predictions can then be compared with the actual value of the test dataset to evaluate the performance of the generated model. Machine learning is useful when working with large and complex data, as well as to support clinical decisions. It can be used to diagnose and predict oral health conditions and personalize prescriptions.

Machine learning has mostly been applied in the medical field up to this point, and not much in adult dental research. It has not, however, been applied to create child caries risk models [20,21]. Utilizing the most pertinent user- and kid-friendly variables from biological, environmental, and socioeconomic factors, we suggest using machine learning to identify children (0–5 years old) at high risk for dental caries. Early identification of this group of kids enables more effective and focused evidence-based preventive treatments, which in turn lowers the potential consequences. The main objective of this study is to investigate dental caries risk among children under 5 and find the potential approach(es) to lower the risk of dental caries in high-risk individuals using ML and personal prescriptions. The model used in this study serves as both a risk and prediction model by identifying the risk variables that contribute to the development of dental caries and predicting who is at risk in order to facilitate prevention and treatment. This methodology is simple to implement and can be applied daily in dental clinics or research investigations.

## 2. Materials and Methods

For this study, we used data from a dental clinic that runs three examination sessions per week for paediatric dental patients. Since these data are completely anonymous, an application for ethical approval was not required. Parents/carers, however, signed a consent form after having understood the pros and cons of participating and their right to drop out from the study.

### 2.1. Data Compilation

The caries risk assessment form (0–6 years) from the American Dental Association (ADA) [22] was used and altered according to the district the study was conducted in. Based on previous research, smoking of parents/carers was added to the questionnaire.

From all patients invited to participate in this research project, information was obtained from a total of 780 patients. Of these, 600 had dental caries and 180 were caries-free. The mean age of participant children in this study was 3.8 years old. All children were examined by a single operator (specialist paediatric dentist) who also filled in the questionnaires and subsequently recorded the data. According to the inclusion criteria for the study, all children 5 years old and under attend a dental examination. Children with medical disabilities affecting oral hygiene habits were excluded from the study because this might interfere with a dental examination.

The class variable of interest for this research project was caries risk assessment for children 5 years old and under. It was a Boolean class that had yes or no answers to indicate the presence or absence of one or more caries based on a clinical examination by a specialist. Dental caries is defined as a biofilm-mediated, diet-modulated, multifactorial, non-communicable, dynamic disease process caused by an ecological dysbiosis between the host and oral biofilms that results in localised destruction of susceptible dental hard tissues over time [18,23,24,25]. This research focuses on assessing the risk of tooth decay in children five years of age and younger because it is believed that controlling tooth decay during this period can help prevent more oral problems in adulthood so that it can be treated or prevented [26].

### 2.2. Supervised Classification

In this study, demographic and clinical characteristics of participants in terms of mean, standard deviation, frequency, and proportion were examined. Machine learning methods were used to classify caries risk of chid dental patients. In total, there are 780 instances and 17 features in the dataset collected for this study. All features were included to define their relative importance based on their F-scores. F-score measures the accuracy of a model on a scale of 0 to 1 (with 0 being the worst and 1 being the best) and determines feature importance based on how often that feature is considered. Features with a higher F-score are likely to play a greater role in predicting dental caries. Datasets were randomly assigned to training and test sets, with 80% used for training and 20% for testing.

In this study, several supervised machine learning methods were used, including Logistic Regression, Extreme Gradient Boosting (XGBoost), Random Forest, Decision Tree, K-Nearest Neighbours (k-NN), and Support Vector Machine (SVM). In traditional medical studies, Logistic Regression is usually applied, and therefore, it was used in this study. Other techniques have been selected because of their tolerance to overfitting, their ability to accurately model nonlinear relationships, ease of implementation in medical applications, and their acceptance in the machine learning communities [20]. Logistic Regression is a linear algorithm used to predict the probability of a target variable. The essence of the target or dependent variable is dichotomous. It means that there would be only two possible classes [27]. Extreme Gradient Boosting (EGB) is a tree-based algorithm and shows the same behaviour as a standard linear regression in that it produces a prediction model in the form of an ensemble of weak prediction models [28]. Random Forest is a linear combination of decision trees that creates decision trees on data samples and then derives the prediction from each of them. In the end, this algorithm selects the best solution by means of voting. Random Forest is an ensemble method and is better than a single decision tree as it diminishes the overfitting by averaging the result [29]. A decision tree is a technique for prediction modelling. This technique applies a predictive model to go from observations about an item represented in the branches to conclusions about the target value shown in the leaves [30]. K-Nearest Neighbours is a non-parametric classification technique where the input consists of the k closest training examples in the dataset [31]. Support Vector Machine is a linear classification model which can solve linear and non-linear problems. In essence, SVM is an algorithm that takes data as input and classifies them, if possible, using a line or hyperplane [32].

## 3. Results

In this section, at first, the dataset is explained. In the second and third steps, the classification with three and two types of risks are described, respectively. Finally, the result of classifications with a K-fold Cross-Validation method is investigated.

### 3.1. Data Compilation

**Figure 1:**
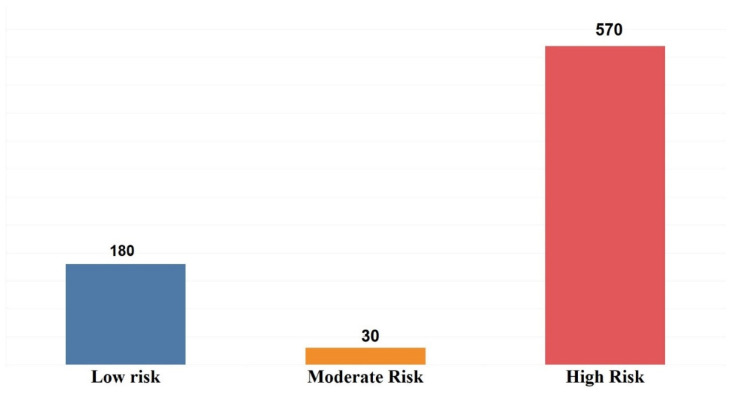
Number of patients in three levels of Low, Moderate, and High Risk.

**Figure 2:**
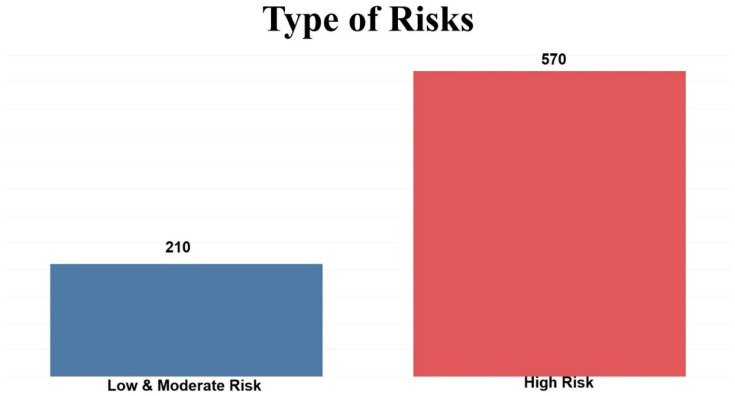
Number of patients in two levels of Low and Moderate Risk, and High Risk.

This dataset consists of 780 records and 17 variables. One of the variables is classified with three values, namely, Low, Moderate, and High Risk. According to Table 1, it is clear that all variables are categorical. The contribution of each variable is represented by the number and percentage for every value. Two experiments were carried out using the dataset. The dataset in the first experiment was divided into three different groups: 180 Low Risk, 30 Moderate Risk, and 570 High Risk. In the second experiment, the dataset was divided into two groups: 210 Low and Moderate Risk, and 570 High Risk. Figure 1 and Figure 2 show the distribution of risks in the first and second experiments.

### 3.2. Supervised Classification

#### 3.2.1. Three Different Risk Levels

**Figure 3:**
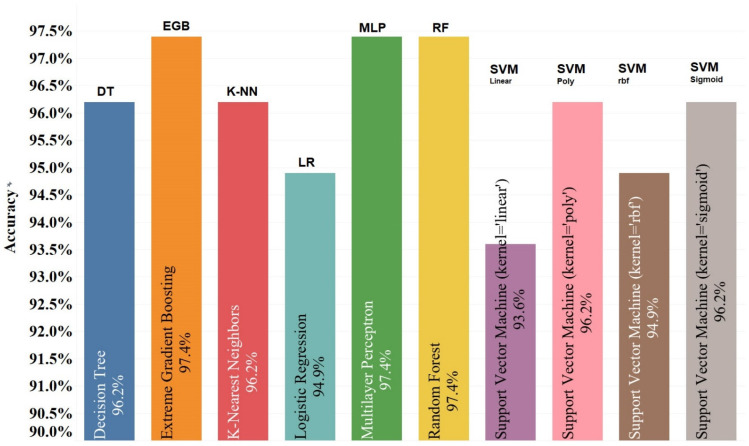
Accuracy of classifiers for 3 different types of risks with the Leave-One-Out Cross-Validation method.

These data are modelled in seven different ways. It should be noted that the Support Vector Machine algorithm is modelled with four different kernels. The Leave-One-Out method is used to evaluate them. For this purpose, the accuracy parameter is considered for the evaluation of the results. Since this variable does not represent the results correctly, it is necessary to consider other variables as well. Three variables, precision, recall, and F1-score, were used to better evaluate the performance of the machine learning algorithms. According to the accuracy, EGB Multilayer Perceptron and Random Forest have the best results, with 97.4%, while the Support Vector Machine with linear kernel has the worst results, with 93.6%. Figure 3 shows the accuracy for the ten different models used for this study.

Comparing the parameters precision, recall, and F1-score, we see that these values are not satisfactory for the Moderate Risk group. In Table 2, the worst results are plotted in red, the middle results are plotted in white, and the best results are plotted in light and dark green. As shown in the graph, even in the cases where the best accuracy is obtained, the parameters that represent the details of the model range from 0 to 67%. This means that if we are going to recognize children who are at risk of dental caries in the Moderate Risk category, the best models are 17% better than tossing coins. The reason for this problem is the low number of records related to the middle class. The number of records belonging to this class is three, as shown in Figure 1. To solve this problem, we added Moderate Risk records to the Low Risk records and rebuilt the models with the new labels.

#### 3.2.2. Two Different Risk Levels

**Figure 4:**
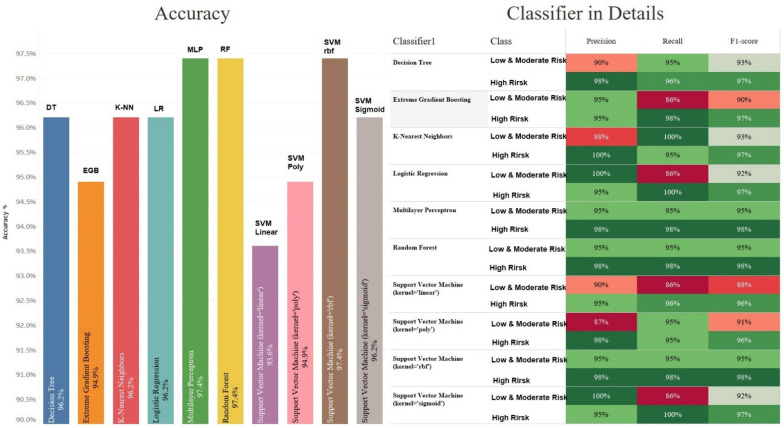
Accuracy of classifiers for 2 different types of risks with the Leave-One-Out Cross-Validation method.

As in the previous section, we trained and evaluated the models. In addition, we used K-fold Cross-Validation to evaluate the models. The value of K in this experiment is 5. After the changes were applied, Multilayer Perceptron and Random Forest had the best accuracy, with 97.4%, as in the previous section. This time, however, Support Vector Machine with Kernel RBF (with an accuracy of 97.4%) was better than Extreme Gradient Boosting (with 94.9% accuracy). The Support Vector Machine model still has the worst accuracy, with a linear kernel at 93.6% accuracy. These results are shown in Figure 4.

Comparing the details of the results obtained for the classes, we can see that the values of the precision, recall, and F1-score are often much better than previously obtained (see Table 2). Table 3 shows that the worst values for precision, recall, and F1-score are 87%, 86%, and 88%, respectively.

K-fold Cross-Validation is used to ensure the stability of the results and to evaluate the models more accurately. As shown in Table 4, the best and worst accuracy are the same for all models. Therefore, to evaluate the stability of the models, the mean and standard deviation are investigated. Usually, the best answer is for the model with the lowest standard deviation and the highest average. In this table, the highest mean value is related to SVM (kernel = ‘sigmoid’) with an average accuracy value of 96.25%, and the lowest standard deviation with a value of 6.58 belongs to the SVM model (kernel = RBF). Although the size of the dataset is small, this is fairly common in medical data, and the quality of the data means that the results obtained for these models are extraordinary. For all of the models, the accuracy range (between 92.25% and 96.25%) is such that they should all be reliable in most cases.

## 4. Discussion

Dental caries is the most common dental disease in children. If left untreated, minor dental caries progresses into deeper tooth structures involving the pulp of teeth and causes pain, discomfort and infection. Therefore, timely and accurate diagnosis is vital in the prevention and treatment of tooth decay and the future oral health of young patients.

Caries risk assessment is an important part of a dental examination session, and many risk assessment models have been introduced to detect those at risk for dental caries and highlight the most important risk factors [5,17].

This study was conducted to provide an accurate model to assess the caries risk of 0–5-year-old dental patients by using machine learning modelling by collecting data from caries-free patients and those with dental caries. Machine learning techniques used for caries risk assessment were supervised learning techniques to permit simultaneous analysis and comparisons of features in both caries-active and caries-free subjects in order to represent a predictive model. We used the ADA caries risk assessment form with minor changes and applied different machine learning models.

Across all methods, present dental caries, consumption of sugary foods/drinks, not attending regular dental visits, parents’/carers’ low socioeconomic level, and low fluoride exposure were among the contributing factors to high caries risk in a patient. This agrees with previous studies using caries risk assessment tools [10,13].

Most of our other contributors to caries risk were consistent with previous research that has identified past caries experience and biological and socioeconomic factors as important features in children with dental caries. In contrast to previous research, we found that parental smoking and having medical conditions did not put children at higher risk for dental caries. This may be due to the low number of patients and those with smoker parents in this pilot study and may need to be revised in future research [12,33,34,35]. Nevertheless, a number of credible studies [14,15,16] indicate that children whose parents smoke are more likely to have tooth decay. As a result, we want to look into this issue more thoroughly in the subsequent study, which will cover a larger geographic area and more data.

The use of machine learning not only helped in the identification of risk factors for caries in children, but also helped generate computer algorithms able to consider combinations of variables. The classifier performance is almost unbiased, and, for this reason, it is generalizable. This makes it a promising source of subject-specific information and gives it potential to have an impact on the prediction of caries risk and classification and help in the early detection of dental caries. This was achieved by applying training and test datasets instead of using all the data to merely analyse attributes. Obviously, applying all the data to generate a predictive model would likely lead to bias in modelling, which is called overfitting from an AI point of view.

We used our collected data and applied multiple machine learning methods to identify the most accurate model related to caries risk assessment in children. The main datasets were divided into three classes: Low Risk, Moderate Risk, and High Risk. It is noteworthy that there is a small amount of data in the Moderate Risk class; therefore, modelling was conducted in two ways, with three classes and with two classes. In the three-class method, the data were modelled as Low Risk, Moderate Risk, and High Risk, while in the two-class method, the data of the Low and Moderate Risk classes were merged.

We focused on finding and targeting high-caries-risk children via dental examinations at an early stage in life, leading to targeting high-risk groups for strict prevention measures. Ten different machine learning modelling techniques and two assessment methods (Leave-One-Out and K-fold) were used. The best performing machine learning models were MLP, RF, and SVM (kernel = RBF), which most accurately classified the presence of risk with an accuracy above 97%. According to the values obtained for precision, recall, and F1-score parameters, which are presented in the Results section, these values indicate the quality of implementation and robustness of the methods.

This study was not without limitations. Firstly, data collection was performed during the COVID-19 pandemic when most families did not attend routine dental check-up appointments. It was, therefore, very difficult to collect caries-free data unless they attended for a viral manifestation, etc. Secondly, taking oral microflora samples and sending them to the lab for bacterial count could cause possible COVID-19 cross-infection; thus, this was omitted from the questionnaire. Thirdly, data were collected from subjects in Iran and may not be representative of other countries, especially when it comes to diet and social factors. Lastly, due to the fact that this was a pilot study, the size of the dataset was limited (with only 780 records). As such, the results of the study may not translate well to a larger dataset.

## Figures and Tables

**Table 1:** Demographic characteristics (N = 780).

Categorical Variables	N	%
***Fluoride exposure***		
*Yes*	180	23.08%
*No*	600	76.92%
***Sugary foods/drinks***		
*Yes*	550	70.51%
*No*	230	29.49%
***Regular dental visits***		
*Yes*	330	42.31%
*No*	450	57.69%
***Special needs***		
*Yes*	10	1.28%
*No*	770	98.72%
***Chemo/radiotherapy***		
*Yes*	0	0.00%
*No*	780	100.00%
***Eating disorders***		
*Yes*	0	0.00%
*No*	780	100.00%
***Medications reducing salivary flow***	290	16.48%
*Yes*	0	0.00%
*No*	780	100.00%
***Cavitated/non-cavitated***		
*Cavitated*	600	76.92%
*Non-cavitated*	180	23.08%
***Carious lesion (visual/radiographically)***		
*Visual*	600	76.92%
*Radiographically*	180	23.08%
***Teeth extracted due to caries within the past 36 months***		
*Yes*	250	32.05%
*No*	530	67.95%
***Visible plaque***		
*Yes*	330	42.31%
*No*	450	57.69%
***Unusual tooth morphology that causes plaque retention***		
*Yes*	0	0.00%
*No*	780	100.00%
***Proximal restorations***		
*Yes*	0	0.00%
*No*	780	100.00%
***Dental/orthodontic appliances***		
*Yes*	0	0.00%
*No*	780	100.00%
***Parents’/carers’ education***		
*High*	430	55.13%
*Medium*	350	44.87%
*Low*	0	0.00%
***Parents’/carers’ monthly income***		
*High*	390	50.00%
*Medium*	390	50.00%
*Low*	0	0.00%

**Table 2:** Classifiers in detail in terms of precision, recall, and F1-score for 3 different types of risks.

Classifier	Class	Precision (%)	Recall (%)	F1-Score (%)
**Decision Tree**	High Risk	98	96	97
	Moderate Risk	50	67	57
	Low Risk	100	100	100
**Extreme Gradient Boosting**	High Risk	98	98	98
	Moderate Risk	67	67	67
	Low Risk	100	100	100
**K-Nearest Neighbour**	High Risk	98	96	97
	Moderate Risk	50	67	57
	Low Risk	100	100	100
**Logistic Regression**	High Risk	94	98	97
	Moderate Risk	0	0	0
	Low Risk	100	100	100
**Multilayer Perceptron**	High Risk	98	98	98
	Moderate Risk	67	67	67
	Low Risk	100	100	100
**Random Forest**	High Risk	94	96	96
	Moderate Risk	0	0	0
	Low Risk	100	100	100
**Support Vector Machine** **(kernel = Linear)**	High Risk	98	96	97
	Moderate Risk	50	67	57
	Low Risk	100	100	100
**Support Vector Machine** **(kernel = Poly)**	High Risk	94	96	96
	Moderate Risk	0	0	0
	Low Risk	100	100	100
**Support Vector Machine** **(kernel = rbf)**	High Risk	98	96	97
	Moderate Risk	50	67	57
	Low Risk	100	100	100
**Support Vector Machine** **(kernel = Sigmoid)**	High Risk	95	100	97
	Moderate Risk	0	0	0
	Low Risk	100	100	100

**Table 3:** Classifiers in detail in terms of precision, recall, and F1-score for 2 different types of risks.

Classifier	Class	Precision (%)	Recall (%)	F1-Score (%)
**Decision Tree**	Low & Moderate Risk	90	95	93
	High Risk	98	96	97
**Extreme Gradient Boosting**	Low & Moderate Risk	95	86	90
	High Risk	95	98	97
**K-Nearest Neighbour**	Low & Moderate Risk	88	100	93
	High Risk	100	95	97
**Logistic Regression**	Low & Moderate Risk	100	86	92
	High Risk	95	100	97
**Multilayer Perceptron**	Low & Moderate Risk	95	95	95
	High Risk	98	98	98
**Random Forest**	Low & Moderate Risk	95	95	95
	High Risk	98	98	98
**Support Vector Machine** **(kernel = Linear)**	Low & Moderate Risk	90	86	88
	High Risk	95	96	96
**Support Vector Machine** **(kernel = Poly)**	Low & Moderate Risk	87	95	91
	High Risk	98	95	96
**Support Vector Machine** **(kernel = rbf)**	Low & Moderate Risk	95	95	95
	High Risk	98	98	98
**Support Vector Machine** **(kernel = Sigmoid)**	Low & Moderate Risk	100	86	92
	High Risk	95	100	97

**Table 4:** Mean, best, and worst accuracy and standard deviation of classifiers for 2 different types of risks with the K-fold Cross-Validation method (k = 5).

Classifier	Mean	Standard Deviation	Best	Worst
Decision Tree	93.58%	8.04	100.00%	81.25%
Extreme Gradient Boosting	94.92%	7.3	100.00%	81.25%
K-Nearest Neighbours	92.25%	7.4	100.00%	81.25%
Logistic Regression	94.92%	7.3	100.00%	81.25%
Multilayer Perceptron	94.92%	7.3	100.00%	81.25%
Random Forest	94.92%	7.3	100.00%	81.25%
Support Vector Machine (kernel = ‘linear’)	93.58%	8.04	100.00%	81.25%
Support Vector Machine (kernel = ‘rbf’)	93.58%	6.58	100.00%	81.25%
Support Vector Machine (kernel = ‘poly’)	93.58%	8.04	100.00%	81.25%
Support Vector Machine (kernel = ‘sigmoid’)	96.25%	7.5	100.00%	81.25%

## Data Availability

The corresponding author or principal investigator can provide the data used in this study upon request. Due to privacy and ethical concerns, the data are not available to the general public.
